# The advantages of intermediate-tier, inter-optometric referral of low risk pigmented lesions

**DOI:** 10.1111/opo.12413

**Published:** 2017-10-17

**Authors:** Angelica Ly, Lisa Nivison-Smith, Michael Hennessy, Michael Kalloniatis

**Affiliations:** 1 Centre for Eye Health, Sydney, NSW Australia; 2 School of Optometry and Vision Science, UNSW Sydney, Sydney, NSW Australia; 3 Department of Ophthalmology, Prince of Wales Hospital, Sydney, NSW Australia

**Keywords:** choroidal naevus, collaboration, pigmented lesions, shared-care

## Abstract

**Purpose:**

Pigmented ocular lesions are commonly encountered by eye-care professionals, and range from benign to sight or life-threatening. After identifying a lesion, the primary care professional must establish the likely diagnosis and decide either to reassure, to monitor or to refer. The increasing use of ocular imaging technologies has contributed to an increase in the detection rate of pigmented lesions and a higher number of referrals, which may challenge existing pathways of health-care delivery. Specialist services may be over-burdened by referring all patients with pigmented lesions for an opinion, while inter-optometric referrals are underutilised. The aim of this study was to describe the referral patterns of pigmented lesions to an optometry led intermediate-tier collaborative care clinic.

**Methods:**

We performed a retrospective review of patient records using the list of patients examined at Centre for Eye Health (CFEH) for an initial or follow up pigmented lesion assessment between the 1/7/2013 and the 30/6/2016. Analysis was performed on: patient demographic characteristics, the referrer's tentative diagnosis, CFEH diagnosis and recommended management plan.

**Results:**

Across 182 patient records, the primary lesion prompting referral was usually located in the posterior segment: choroidal naevus (105/182, 58%), congenital hypertrophy of the retinal pigment epithelium (CHRPE; 11/182, 6%), chorioretinal scarring (10/182, 5%) or not specified (52/182, 29%). Referrals described a specific request for ocular imaging in 25 instances (14%). The number of cases with a non-specific diagnosis was reduced after intermediate-tier care assessment (from 29% to 10%), while the number of diagnoses with less common conditions rose (from 2% to 21%). There was a 2% false positive referral rate to intermediate-tier care and a first visit discharge rate of 35%. A minority required on-referral to an ophthalmologist (22/182, 12%), either for unrelated incidental ocular findings, or suspicious choroidal naevi. Conditions most amenable to optometric follow up included: 1) chorioretinal scarring, 2) choroidal naevus, and 3) CHRPE.

**Conclusions:**

Intermediate-tier optometric eye-care in pigmented lesions (following opportunistic primary care screening) has the potential to reduce the number of cases with non-specific diagnoses and to increase those with less common diagnoses. The majority of cases seen under this intermediate-tier model required only ongoing optometric surveillance.

## Background

Pigmented ocular lesions may be defined as any melanocytic abnormality of the eye or associated tissues and includes intraocular tumours (benign, indeterminate or malignant lesions of the uvea, retina, retinal pigment epithelium or optic nerve), metastases, scarring or hyperplasia associated with degenerative, inflammatory or neovascular disease. Failure to distinguish benign pigmented lesions from potentially malignant conditions, such as choroidal melanoma, can result in delays in care, suboptimal treatment outcomes and a greater need for enucleation.^1^ However, choroidal melanoma is also rare and can be difficult to recognise even by experienced clinicians.^2^ Benign, atypical or suspicious lesions additionally require indefinite, periodic surveillance with intervals set to address the risk for malignant change.^4^ Early identification is key and may be improved with specialist expertise and ocular imaging technologies.^2^

The increasing use of ocular imaging among eye-care practitioners has been associated with an increased detection rate and excessive referrals for pigmented lesions.^3^ The conventional referral pathway for patients with pigmented lesions in many eye-care systems starts with an assessment in primary care, followed by referral to a general ophthalmologist, and then onto a retinal specialist or ocular oncologist as required. However, specialist care may be overburdened by false positive referrals of benign lesions, and inter-optometric referrals are uncommon.^9^ Chronic and/or well controlled conditions may not require ophthalmological management.^11^ Additionally, core competencies for entry level into the optometric profession stipulate that optometrists should have the ability to assess and evaluate the various ocular tissues for the purpose of screening for health or disease.^12^ Similarly, optometrists are trained to recognise clinical situations which do not require intervention and those that necessitate periodic review due to risk of visual or systemic morbidity. Thus, there has been a growing interest in inter-professional collaborative care, especially schemes capitalising on the skills of allied health, to address the needs of an increasingly aged population in developed nations, the expectation of maintaining good health, the wait times to see a specialist, and resource limitations.^8^ Efficiency in eye-care delivery is the goal whereby each patient sees the right professional at the right time. Efficiency might be improved via referral refinement schemes integrating optometrists with a special interest.^15^

In Australia, access to eye-care services is covered at least in part through the national government reimbursement scheme, known as Medicare. Primary eye-care for 24.6 million Australians is provided predominantly by the 5134 practising optometrists across the country, and occasionally by general medical practitioners.^16^ Subsequent secondary or tertiary ophthalmology services may be provided either privately at a cost to the patient (rebated in part by Medicare contingent on a valid referral), or publicly at no cost through public hospitals. However, the latter commonly experience waiting times well in excess of clinical recommendations.^18^ In the major capital cities where 78% and 84% of optometrists and ophthalmologists respectively practice, eye-care access is substantial.^19^ On the contrary, approximately 10% of patients in Australia (especially those living in rural areas) may have never seen an eye-care provider.^20^ By large, optometrists already play a key role in the early detection, prevention and management of ocular and visual disorders. However, the benefits of intermediate-tier, optometry-led eye-care are yet to be widely realised. A working model of this pathway is provided by the Centre for Eye Health (CFEH) located in Kensington, Sydney Australia.

The aim of this study was to describe pigmented lesion referral patterns to this optometry-ophthalmology collaborative care clinic and to quantify the level of diagnostic congruency between primary, community care optometrists and intermediate-tier CFEH care, providing an evidence base regarding the role of optometry in the collaborative care of pigmented lesions. We define intermediate-tier care as an intermediary health care service accessed between primary care, a patient's point of first entry into the health system, and secondary specialist ophthalmological care.

## Methods

### Subject selection

This study was a retrospective record review of patients that presented to CFEH for a pigmented lesion assessment. Informed written consent was obtained in accordance with the Declaration of Helsinki and approved by a Biomedical Human Research Ethics Advisory Panel of the University of New South Wales, Sydney Australia. Inclusion criteria were as follows: (1) the patient presented for an initial or follow up pigmented lesion assessment between the 1/7/2013 and the 30/6/2016, (2) a completed referral form associated with the patient's first attendance for a pigmented lesion assessment was provided, (3) a CFEH report relating to the same first visit was available in the electronic patient record management system and contained a clinical summary and recommended management plan. Cases referred for multiple assessments i.e. lesions or suspected disorders other than the primary pigmented lesion were excluded.

CFEH is an integrated care establishment that provides non-urgent imaging and visual system diagnostic services to patients referred from community eye-care, typically optometric primary care, in Sydney, Australia. The CFEH is operationally co-ordinated by optometrists with a close working relationship with the local health district public ophthalmology service. It offers a broad range of services, at no cost to the patient or the referrer, which are typically only available otherwise through either large private ophthalmology practices or public hospitals. Primary funding of CFEH is provided by the philanthropic organisation, Guide Dogs NSW/ACT, with the intent of reducing the incidence of preventable vision loss via increased access to advanced diagnostic services and the early identification of eye disease. Other funding sources include Medicare billing for service delivery and UNSW Australia.

Under the CFEH clinical model, each patient attendance is typically initiated by the referrer completing a structured referral form which is then reviewed by a CFEH optometrist. A standardised appointment type is arranged and then conducted, followed by a report delivered within one week to the referring professional. The CFEH staff optometrists who conduct the assessments receive additional, on-going training within CFEH. The reports are prepared in consultation with an on-site ophthalmologist as required, who also has ready access to the high resolution imaging results. No face-to-face consultation between the patient and ophthalmologist occurs. Urgent referrals, identified by triage of the requests, are redirected to external ophthalmological care by telephoned recommendation to the referring professional and not seen at CFEH. Details of this protocol have been described in previous peer-reviewed publications^21^ and are also available at the CFEH website (http://www.centreforeyehealth.com.au).

### Subject assessment

All subjects in the study completed an entering questionnaire and other forms providing basic demographic and clinical historical data as well as consent to research. Although this was a retrospective study, as part of standard protocols of the CFEH clinic, the patient is given a consent form on presentation to enlist their general consent for research or teaching purposes. A standardised pigmented lesion clinical assessment is then performed including: (1) entering visual function testing – visual acuity, and contrast sensitivity or perimetry on indication, (2) funduscopy, (3) ocular imaging – including colour fundus photography (Kowa WX 3D non-mydriatic retinal camera, Kowa, http://www.kowamedical.com/), wide-field imaging (Optos Panoramic 200Tx or Optos California, Optos, http://www.optos.com/) optical coherence tomography (Spectralis OCT, Heidelberg Engineering, https://www.heidelbergengineering.com/) and B-scan ultrasonography (Tomey UD 6000A, Tomey, http://www.tomey.com/) where applicable. Following each assessment, a report was forwarded to the referring professional electronically by the examining optometrist, with the report co-signed by a senior peer optometrist or a consultant ophthalmologist. The latter typically occurs in instances where referral to an ophthalmologist is recommended in the management plan. Periodically, CFEH arranges for a consultant ophthalmologist to review randomly selected reports signed by senior peer optometrists.

### Data extraction and statistical analysis

Demographic, referral and CFEH report data relating to the patient's first attendance for a pigmented lesion assessment were extracted from the patient record management system (VIP.net, Houston Medical, http://www.houstonmedical.net). Referrals were assessed by AL for the suspected diagnosis, any details provided of the lesion history, and any specific request for ocular imaging. The final diagnosis and recommended management plan were then extracted from the CFEH report. The referrer's suspected diagnosis and CFEH diagnosis were classified into one of six categories – choroidal naevus, congenital hypertrophy of the retinal pigment epithelium (CHRPE), chorioretinal scar, other, normal, or non-specific. Diagnoses comprising less than 5% of the total dataset (nine cases) were grouped together as other. In cases of co-morbidities, only the primary diagnosis was coded using information specified under other fields (primary reason for referral and pertinent exam findings). Similarly, the final CFEH diagnosis was coded into the category with the greatest influence on the recommended management plan. For recalled patients, follow up consultation dates were also extracted.

Figures were created using GraphPad Prism (Version 6; www.graphpad.com). Coded data were analysed using frequency of occurrence and diagnostic congruency was assessed using a contingency matrix. Chi-square analysis, using a *p*-value less than 0.05, was used to test for a statistically significant difference between the diagnoses stipulated on the referral compared to the CFEH report.

## Results

### Patient characteristics

A total of 182 patient records were included in the analysis. Patients were predominantly female (101, 55%), resided in NSW apart from one patient from the ACT, and ranged between 19 and 76 years of age (mean of 48 years). There was no statistically significant difference between genders regarding the suspected diagnosis (*χ*^2^ (4) = 5.75, *p* = 0.22), CFEH diagnosis (*χ*^2^ (5) = 5.68, *p* = 0.34), ongoing management recommendation (*χ*^2^ (2) = 1.23, *p* = 0.54) or compliance with follow up (*χ*^2^ (2) = 0.70, *p* = 0.71). Referrals were provided by 69 primary care clinics across Sydney. This approximated 0.04% of the referrers’ total patient base (assuming a 46 week year and an average of 45 patients per week across the three year period).^23^

### Primary reason for referral

**Figure 1:**
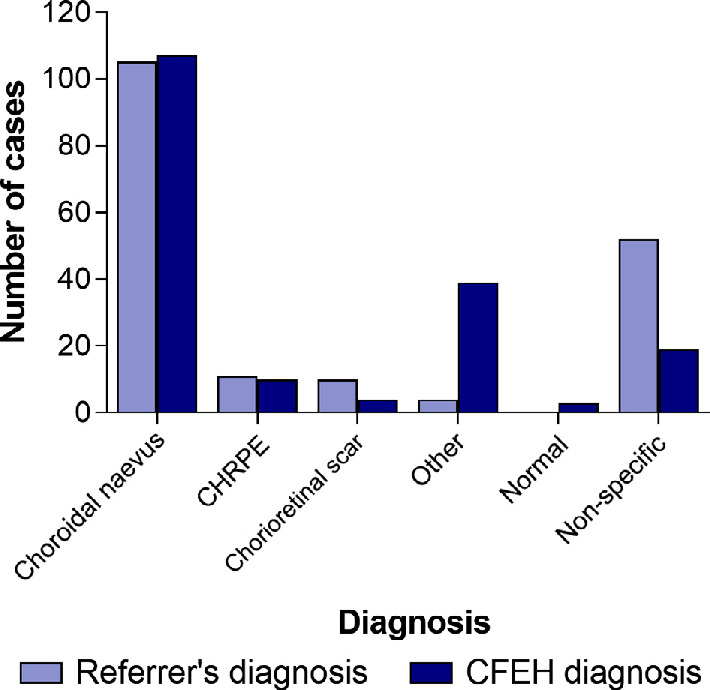
Distribution of referral and CFEH diagnoses.

The distribution of tentative diagnoses stipulated on the initial referral is shown in *Figure* 1. The primary lesion was usually located in the posterior rather than the anterior segment i.e. choroidal naevus, CHRPE or chorioretinal scarring. Choroidal naevus was the suspected diagnosis in 105/182 (58%) and the CFEH diagnosis in 107/182 (59%). Referrals describing a suspected diagnosis of chorioretinal scarring typically also specified toxoplasmosis (7/10). The ‘other’ diagnoses included atrophy (two) and drusen (two). Almost 30% of referrals (52/182) did not nominate a specific diagnosis.

Forty-five referrals (25%) noted a history of the lesion by the referrer, either using their records, clinical observation or through direct patient questioning, and 25 (14%) explicitly included comments on the value of ocular imaging. The latter was usually a non-specific comment (13/25, 52%); there were five explicit requests for optical coherence tomography, five for ultra-widefield imaging, three for photography and one for ultrasonography. Two referrals specifically requested two imaging modalities.

### Patient diagnoses on referral and after evaluation at CFEH

*Table* 1 presents the diagnostic congruency of the referred cases. Despite a high degree of diagnostic congruency for choroidal naevus and CHRPE, the distribution of tentative diagnoses provided by the referrer differed significantly from the CFEH diagnosis (*χ*^2^ (20) = 157.03, *p* < 0.0001). The number of diagnoses categorised into ‘other’ i.e. diagnosed with an uncommon condition rose from 4 to 39 (2% to 21%) following evaluation at CFEH, and included: chorioretinal atrophy (eight instances), and two instances each of drusen, focal choroidal excavation, iris naevus, ocular melanocytosis, pigment epithelial detachment, RPE hyperplasia, RPE window defect and vitreoretinal tuft. Diagnoses occurring once out of the 182 cases have not been listed.

**Table 1:**
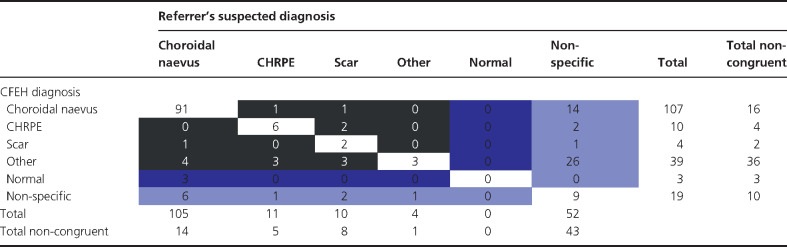
Correlation matrix showing diagnostic congruency between primary care and the Centre for Eye Health shared-care; congruent cases are shaded white and misdiagnoses in black. Cases diagnosed by the referrer or CFEH as normal i.e. with no apparent defect are indicated in dark blue, while the light blue shading denotes any cases with a non-specific diagnosis

The number of cases without a specific diagnosis was reduced by approximately two-thirds (29% to 10%). Of the 52 cases referred without a diagnosis, 43 (83%) were provided with a specific diagnosis following assessment at CFEH – usually other (26), followed by choroidal naevus (14), CHRPE (2) or chorioretinal scarring (1). Nineteen cases (10%) of the total cohort could not be provided with a specific diagnosis following CFEH assessment despite half undergoing ophthalmological review (ten reports). Cases were seldom (15/182, 8%) misdiagnosed as a completely different ocular condition. Three instances were referred to CFEH due to suspected choroidal naevus though found by CFEH not to have any pigmented lesions, (3/182, 2% false positive referral rate).

### Recommended ongoing care plan

**Figure 2:**
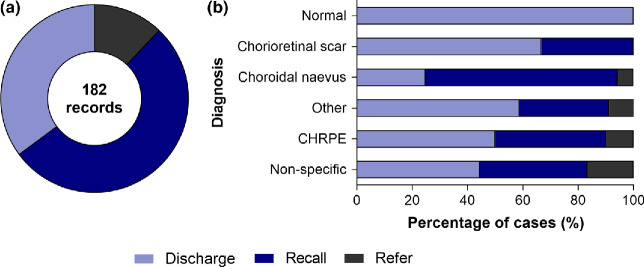
Overview of the CFEH recommended management plan across 182 records. Discharge describes normal or low risk patients where CFEH recommended ongoing review with the referring professional only. Recall indicates the group of cases at risk of disease progression that were recalled for ongoing surveillance at CFEH. Refer describes the group recommended referral to an ophthalmologist for specialist expertise or treatment. (a) Using the total cohort of 182 records, 35% were recommended discharge, 53% were suitable for CFEH review, while the remaining 12% were advised to seek ophthalmological opinion. (b) The various diagnostic categories showing a breakdown of their CFEH recommended management plan, excluding nine cases referred for incidental ocular findings.

The CFEH report most frequently recommended recall for CFEH review (96/182, 53%), followed by discharge back to community care (64/182, 35%), or referral to an ophthalmologist (22/182, 12%; *Figure* 2*a*). Of the 96 cases recommended review at CFEH, 61 were seen at CFEH again at least once for a subsequent follow up appointment (64% compliance). Presentations suitable for optometric management based on the CFEH report recommendations (discharge or recall to CFEH, *Figure* 2*b*) included, in decreasing order: (1) normal, (2) chorioretinal scarring, (3) choroidal naevus, and (4) CHRPE.

The cases recommended referral to an ophthalmologist were diagnosed with choroidal naevus (six cases), peripheral retinal changes (retinal detachment, retinal holes, vitreoretinal traction; three cases) and CHRPE (one case). Three cases did not have a specific diagnosis though the CFEH report specified signs of subretinal fluid in the clinical summary. The remaining nine instances were recommended referral to an ophthalmologist due to unrelated incidental ocular findings – retinal vascular changes, established pituitary tumour related visual field loss, glaucoma, cataract, retrograde degeneration, diabetic retinopathy, optic neuropathy, vitreoretinal traction and shallow anterior chamber angles.

## Discussion

The appearance, histopathology and differential diagnosis of pigmented ocular lesions has been reviewed extensively elsewhere.^24^ In summary, benign pigmented fundus lesions are often asymptomatic and may not be reliably diagnosed or distinguishable from more sinister pathologies. This report demonstrates that the use of intermediate-tier care optometry may aid in the appropriate referral of pigmented lesions. The number of cases without a specific diagnosis decreased by two-thirds following evaluation at CFEH, and these cases were usually found to have an uncommon or more recently characterised clinical entity due to the emergence of multimodal imaging, such as focal choroidal excavation. These findings are consistent with reports of unusual lesions that may simulate melanoma and also support the utility of ocular imaging (including photo-documentation and echography^28^) and intermediate-tier optometry-ophthalmology collaborative care in academic affiliated centres for determining uncommon or atypical diagnoses.^21^

CFEH represents a relatively unique concept among eye-care. It provides timely access to multimodal imaging via 16 imaging and visual function services in one establishment, at no cost to the referrer or patient. Although imaging is integral to the better diagnosis provided by CFEH, additional contributing factors include: clear communication between the referring and intermediate care party (facilitated by a standardised referral form and electronic reporting), judicious screening of incoming referrals before an appointment is booked, rapid access to ophthalmological opinion, evidence based practice and optometric staff with specific training in the diagnosis of pigmented lesions that is beyond the basic competencies expected of Australian optometrists.

### The emergence of ocular imaging

The bulk referral base to CFEH derives from primary care Australian optometrists. Optical coherence tomography, stereoscopic mydriatic slit lamp funduscopy and optic disc photography are performed by approximately 58%, 95%, and 96% of primary care optometrists respectively.^23^ The number of practices with optical coherence tomography in Australia was formerly estimated at 30% in 2012,^29^ and this trend toward increasing investment in ocular imaging equipment by optometrists is also occurring in other nations, such as the UK.^30^ Thus, and due in part to this widespread emergence of routine ocular imaging and retinal photography, primary eye-care professionals are today more than ever before likely to encounter pigmented lesions of a suspicious nature.

Although conventional fundus photography typically captures a field of view in the order of 45 degrees centred on the posterior pole, ultra-widefield scanning laser ophthalmoscopy has become commercially available and boasts a remarkable ability to capture 200 degrees (or approximately 80%) of the fundus in a single image.^6^ It can be performed undilated and routine, non-mydriatic photo-documentation of pigmented fundus lesions, whether for screening or following first detection of ocular disease, might also be attractive to the clinician for patient education, serial surveillance or from a medicolegal perspective. Ultra-widefield imaging assisted dilated fundus examination has also been associated with a statistically significant advantage for the detection of choroidal naevi, compared to traditional dilated fundus examination alone.^7^

Other technologies, such as spectral domain optical coherence tomography provide a unique, if not indispensable, cross-sectional and high-resolution representation of the optical properties of a pigmented lesion.^31^ For example, it permits visualisation of the intrinsic reflectivity of choroidal naevi and overlying microstructural changes to the retina and/or retinal pigment epithelium, including drusen, atrophy, hypertrophy, fibrous metaplasia or an RPE trough.^33^ For choroidal melanoma, optical coherence tomography can be vital in the detection of subretinal fluid and the overlying retinal characteristics considered to indirectly stage the rate of tumour growth (whereby a chronic optical coherence tomography pattern is signified by intraretinal cysts and/or atrophy).^34^

Finally, fundus autofluorescence imaging may, in brief, provide critical insight into the autofluorescence characteristics of the pigmented lesion itself as well as other associated changes, especially changes involving the RPE, such as drusen or subretinal fluid.^35^ Most pertinently, fundus autofluorescence imaging can enhance the ability to visualise orange pigment (lipofuscin) on a tumour's surface associated with malignancy, which colocalises with distinct hyper-autofluorescence.^36^ In contrast, large drusen on the surface of a choroidal naevus might appear as discrete spots of hyper-autofluorescence, while subretinal fluid typically displays a mildly hyper-autofluorescent leading edge or gravitational effect.^37^ Dark, hypo-autofluorescent areas associated with pigmented posterior eye lesions typically evolve over time, coinciding with degeneration and/or atrophy.^37^

Overall, the additional information gleaned from ancillary imaging of pigmented fundus lesions (either accessed in house or through referral to services such as CFEH) might be difficult to acquire using ophthalmoscopy or fundus photography alone and may subsequently be usefully applied to clinical management decisions. The management of pigmented lesions might, in addition, benefit from referral refinement or audit schemes such has been performed in conditions like glaucoma,^9^ repeat-measure schemes,^41^ validated referral guides,^42^ facilities associated with a large, specialist centres^28^ and open-source clinical resources.^42^ A tele-consultation or “virtual clinic” approach (both synchronous and asynchronous) may also be helpful.^8^

Of the 12% of cases seen at CFEH that required on-referral for ophthalmological opinion, no cases of choroidal melanoma were found, although seven cases had an atypical lesion appearance (choroidal naevi and CHRPE). Although population and lesion differences preclude direct comparison of the diagnostic accuracy rate to other studies,^3^ our data supports a high level of diagnostic congruency between primary and intermediate-tier care optometry as well as a low overall false positive rate. This represents a high level of lesion awareness, appropriate detection and referral practices and improved diagnostic techniques among the practising clinicians utilising the centre.^25^ Similarly, substantial agreement in the clinical management decisions regarding melanocytic fundus lesions by optometrists compared with ophthalmological opinion has been reported elsewhere.^8^

### Study limitations

This study was limited by its retrospective design and the range of cases referred to CFEH, with a potential bias toward non-malignant posterior segment lesions. Choroidal melanoma, metastases or urgent presentations are likely referred directly to ophthalmologists. Indeed the distribution of pigmented fundus lesions most amenable to optometric care that are commonly referred to CFEH corresponds with the approximate prevalence of these lesions in a general population: choroidal naevi is most common, with a 10% prevalence in a cohort of healthy college students.^6^ In contrast, the prevalence of CHRPE has been reported at 1.2%.^43^

Paediatric presentations^44^ are not commonly referred to CFEH and thus were not reported. CFEH diagnoses were also determined by clinical examination and imaging, and not confirmed by histopathology. Despite the two clinician CFEH report review system, misdiagnoses are still possible and the effect of identifying subtle clinical signs such as lipofuscin^42^ was also beyond the scope of this study. Finally, the patients with pigmented lesions are not managed at CFEH and remain the responsibility of the referring professional. Consequently, detailed data on patient outcomes or follow-up outside CFEH was not available.

In conclusion, this study describes a range of pigmented lesions amenable to primary and intermediate-tier eye-care and shows a high level of diagnostic congruency between both groups. It also illustrates the potential role of intermediate-tier care in reducing the number of pigmented lesion cases without a specific diagnosis.

## References

[1] Damato EM & Damato BE. Detection and time to treatment of uveal melanoma in the United Kingdom: an evaluation of 2,384 patients. *Ophthalmology* 2012; 119: 1582–1589. 22503229 10.1016/j.ophtha.2012.01.048

[2] Law C, Krema H & Simpson ER. Referral patterns of intraocular tumour patients to a dedicated Canadian ocular oncology department. *Can J Ophthalmol* 2012; 47: 254–261. 22687302 10.1016/j.jcjo.2012.03.047

[3] Khan J & Damato BE. Accuracy of choroidal melanoma diagnosis by general ophthalmologists: a prospective study. *Eye (Lond)* 2007; 21: 595–597. 16470216 10.1038/sj.eye.6702276

[4] Shields CL, Furuta M, Berman EL et al. Choroidal nevus transformation into melanoma: analysis of 2514 consecutive cases. *Arch Ophthalmol* 2009; 127: 981–987. 19667334 10.1001/archophthalmol.2009.151

[5] Silva PS, Cavallerano JD, Haddad NM et al. Comparison of nondiabetic retinal findings identified with nonmydriatic fundus photography vs ultrawide field imaging in an ocular telehealth program. *JAMA Ophthalmol* 2016; 134: 330–334. 26795026 10.1001/jamaophthalmol.2015.5605

[6] Gordon-Shaag A, Barnard S, Millodot M et al. Prevalence of choroidal naevi using scanning laser ophthalmoscope. *Ophthalmic Physiol Opt* 2014; 34: 94–101. 24325439 10.1111/opo.12092

[7] Brown K, Sewell JM, Trempe C, Peto T & Travison TG. Comparison of image-assisted versus traditional fundus examination. *Eye Brain* 2013; 5: 1–8. 28539783 10.2147/EB.S37646 PMC5432113

[8] Balaskas K, Gray J, Blows P et al. Management of choroidal naevomelanocytic lesions: feasibility and safety of a virtual clinic model. *Br J Ophthalmol* 2016; 100: 665–670. 26347525 10.1136/bjophthalmol-2015-307168

[9] Baker H, Ratnarajan G, Harper RA, Edgar DF & Lawrenson JG. Effectiveness of UK optometric enhanced eye care services: a realist review of the literature. *Ophthalmic Physiol Opt* 2016; 36: 545–557. 27580754 10.1111/opo.12312

[10] Jamous KF, Jalbert I, Kalloniatis M & Boon MY. Australian optometric and ophthalmologic referral pathways for people with age-related macular degeneration, diabetic retinopathy and glaucoma. *Clin Exp Optom* 2014; 97: 248–255. 24400653 10.1111/cxo.12119

[11] Rowe S, MacLean CH & Shekelle PG. Preventing visual loss from chronic eye disease in primary care: scientific review. *JAMA* 2004; 291: 1487–1495. 15039416 10.1001/jama.291.12.1487

[12] Kiely PM & Slater J. Optometry Australia Entry-level Competency Standards for Optometry 2014. *Clin Exp Optom* 2015; 98: 65–89. 25545949 10.1111/cxo.12216

[13] Shipp MD. Health care changes from a public health perspective: implications for optometry-ophthalmology relations. *Optom Vis Sci* 1997; 74: 1019–1024. 9423993 10.1097/00006324-199712000-00023

[14] Kalloniatis M & Ly C. The role of optometry in collaborative eye care. *Clin Exp Optom* 2016; 99: 201–203. 27178759 10.1111/cxo.12403

[15] Bourne RR, French KA, Chang L, Borman AD, Hingorani M & Newsom WD. Can a community optometrist-based referral refinement scheme reduce false-positive glaucoma hospital referrals without compromising quality of care? The community and hospital allied network glaucoma evaluation scheme (CHANGES). *Eye (Lond)* 2010; 24: 881–887. 19648892 10.1038/eye.2009.190

[16] Optometry board of Australia. Registrant data Reporting period: 1 October 2016 - 31 December 2016. 2016, http://www.optometryboard.gov.au/Policies-Codes-Guidelines.aspx, accessed 30/04/2017.

[17] Australian Bureau of Statistics. Population clock. 2017; http://www.abs.gov.au/ausstats/abs%40.nsf/94713ad445ff1425ca25682000192af2/1647509ef7e25faaca2568a900154b63?OpenDocument, accessed 21/08/2017.

[18] Optometry Australia. Submission to the Senate Select Committee on Health. 2017, http://www.optometry.org.au/advocacy/submissions/, accessed 14/07/2017.

[19] AIHW. Eye health workforce in Australia. 2016, http://aihw.gov.au/publication-detail/?id=60129555186, accessed 14/07/2017.

[20] Keeffe JE, Weih LM, McCarty CA & Taylor HR. Utilisation of eye care services by urban and rural Australians. *Br J Ophthalmol* 2002; 86: 24–27. 11801497 10.1136/bjo.86.1.24 PMC1770984

[21] Ly A, Nivison-Smith L, Hennessy MP & Kalloniatis M. Collaborative care of non-urgent macular disease: a study of inter-optometric referrals. *Ophthalmic Physiol Opt* 2016; 36: 632–642. 27790767 10.1111/opo.12322 PMC5129555

[22] Jamous KF, Kalloniatis M, Hennessy MP, Agar A, Hayen A & Zangerl B. Clinical model assisting with the collaborative care of glaucoma patients and suspects. *Clin Exp Ophthalmol* 2015; 43: 308–319. 25362898 10.1111/ceo.12466

[23] Kiely PM, Cappuccio S & McIntyre E. Optometry Australia Scope of Practice Survey 2015. *Clin Exp Optom* 2017; 100: 260–269. 28295595 10.1111/cxo.12538

[24] Rennie IG. Things that go bump in the light. The differential diagnosis of posterior uveal melanomas. *Eye (Lond)* 2002; 16: 325–346. 12101438 10.1038/sj.eye.6700117

[25] Shields JA, Mashayekhi A, Ra S & Shields CL. Pseudomelanomas of the posterior uveal tract: the 2006 Taylor R. Smith Lecture. *Retina* 2005; 25: 767–771. 16141866 10.1097/00006982-200509000-00013

[26] Traboulsi EI. Pigmented and depigmented lesions of the ocular fundus. *Curr Opin Ophthalmol* 2012; 23: 337–343. 22847029 10.1097/ICU.0b013e32835622b0

[27] Ly A, Nivison-Smith L, Hennessy M & Kalloniatis M. Pigmented lesions of the retinal pigment epithelium. *Optom Vis Sci* 2015; 92: 844–857. 26099061 10.1097/OPX.0000000000000640

[28] Accuracy of diagnosis of choroidal melanomas in the. Collaborative Ocular Melanoma Study. COMS report no. 1. *Arch Ophthalmol* 1990; 108: 1268–1273. 2205183 10.1001/archopht.1990.01070110084030

[29] The Angiogenesis Foundation. Advocating for Improved Treatment and Outcomes for Wet Age-Related Macular Degeneration. A report based on the Australian Wet Age-Related Macular Degeneration Coalition Expert Summit. 2012, https://angio.org/wp-content/uploads/2013/10/au-whitepaper.pdf, accessed 14/07/2017.

[30] Dabasia PL, Edgar DF, Garway-Heath DF & Lawrenson JG. A survey of current and anticipated use of standard and specialist equipment by UK optometrists. *Ophthalmic Physiol Opt* 2014; 34: 592–613. 25160893 10.1111/opo.12150

[31] White UE, Sampson GP, Edwards K, Pritchard N & Efron N. Using optical coherence tomography for the differential diagnosis of a pigmented choroidal lesion. *Clin Exp Optom* 2011; 94: 385–386. 21545649 10.1111/j.1444-0938.2011.00586.x

[32] Shields CL, Mashayekhi A, Materin MA et al. Optical coherence tomography of choroidal nevus in 120 patients. *Retina* 2005; 25: 243–252. 15805899 10.1097/00006982-200504000-00001

[33] Chien JL, Sioufi K, Surakiatchanukul T, Shields JA & Shields CL. Choroidal nevus: a review of prevalence, features, genetics, risks, and outcomes. *Curr Opin Ophthalmol* 2017; 28: 228–237. 28141766 10.1097/ICU.0000000000000361

[34] Espinoza G, Rosenblatt B & Harbour JW. Optical coherence tomography in the evaluation of retinal changes associated with suspicious choroidal melanocytic tumors. *Am J Ophthalmol* 2004; 137: 90–95. 14700649 10.1016/s0002-9394(03)00868-7

[35] Chin K & Finger PT. Autofluorescence characteristics of suspicious choroidal nevi. *Optometry* 2009; 80: 126–130. 19264288 10.1016/j.optm.2008.07.018

[36] Shields CL, Bianciotto C, Pirondini C, Materin MA, Harmon SA & Shields JA. Autofluorescence of choroidal melanoma in 51 cases. *Br J Ophthalmol* 2008; 92: 617–622. 18441171 10.1136/bjo.2007.130286

[37] Kaur G & Anthony SA. Multimodal imaging of suspicious choroidal neoplasms in a primary eye-care clinic. *Clin Exp Optom* 2017. DOI: 10.1111/cxo.12537. [Epub ahead of print] 10.1111/cxo.12537 28370509

[38] Khan S, Clarke J & Kotecha A. Comparison of optometrist glaucoma referrals against published guidelines. *Ophthalmic Physiol Opt* 2012; 32: 472–477. 23009293 10.1111/j.1475-1313.2012.00943.x

[39] Scully ND, Chu L, Siriwardena D, Wormald R & Kotecha A. The quality of optometrists’ referral letters for glaucoma. *Ophthalmic Physiol Opt* 2009; 29: 26–31. 19154277 10.1111/j.1475-1313.2008.00600.x

[40] Cheng J, Beltran-Agullo L, Trope GE & Buys YM. Assessment of the quality of glaucoma referral letters based on a survey of glaucoma specialists and a glaucoma guideline. *Ophthalmology* 2014; 121: 126–133. 24140116 10.1016/j.ophtha.2013.08.027

[41] Kotecha A, Brookes J & Foster PJ. A technician-delivered ‘virtual clinic’ for triaging low-risk glaucoma referrals. *Eye (Lond)* 2017; 31: 899–905. 28211881 10.1038/eye.2017.9 PMC5518844

[42] Hemmerdinger C, Beech M, Groenewald C & Damato B. Validation of an online referral guide for melanocytic fundus lesions. *Ophthalmic Physiol Opt* 2011; 31: 574–579. 21446922 10.1111/j.1475-1313.2011.00830.x

[43] Coleman P & Barnard NA. Congenital hypertrophy of the retinal pigment epithelium: prevalence and ocular features in the optometric population. *Ophthalmic Physiol Opt* 2007; 27: 547–555. 17956359 10.1111/j.1475-1313.2007.00513.x

[44] Maki JL, Marr BP & Abramson DH. Diagnosis of retinoblastoma: how good are referring physicians? *Ophthalmic Genet* 2009; 30: 199–205. 19852578 10.3109/13816810903258837

